# Ovarian cancer ascites proteomic profile reflects metabolic changes during disease progression

**DOI:** 10.1016/j.bbrep.2024.101755

**Published:** 2024-06-13

**Authors:** Diana Luísa Almeida-Nunes, Mariana Nunes, Hugo Osório, Verónica Ferreira, Cláudia Lobo, Paula Monteiro, Miguel Henriques Abreu, Carla Bartosch, Ricardo Silvestre, Ricardo Jorge Dinis-Oliveira, Sara Ricardo

**Affiliations:** a Differentiation and Cancer Group, Institute for Research and Innovation in Health (i3S) of the University of Porto, 4200-135, Porto, Portugal; b Associate Laboratory I4HB, Institute for Health and Bioeconomy, University Institute of Health Sciences—CESPU, 4585-116, Gandra, Portugal; c UCIBIO—Applied Molecular Biosciences Unit, Toxicologic Pathology Research Laboratory, University Institute of Health Sciences (1H-TOXRUN, IUCS-CESPU), 4585-116, Gandra, Portugal; d School of Medicine and Biomedical Sciences (ICBAS), University of Porto, 4050-313, Porto, Portugal; e Proteomics Scientific Platform, Institute for Research and Innovation in Health (i3S) of the University of Porto, 4200-135, Porto, Portugal; f Department of Pathology, Faculty of Medicine from University of Porto (FMUP), 4200-319, Porto, Portugal; g Department of Pathology, Portuguese Oncology Institute of Porto (IPO-Porto), 4200-072, Porto, Portugal; h Department of Medical Oncology, Portuguese Oncology Institute of Porto (IPO-Porto), 4200-072, Porto, Portugal; i Porto Comprehensive Cancer Center Raquel Seruca (PCCC), Portuguese Oncology Institute of Porto (IPO-Porto), 4200-072, Porto, Portugal; j Cancer Biology & Epigenetics Group, Research Center of Portuguese Oncology Institute of Porto (CI-IPO-Porto) / Health Research Network (RISE@CI-IPO-Porto), Portuguese Oncology Institute of Porto (IPO-Porto), 4200-072, Porto, Portugal; k Life and Health Sciences Research Institute (ICVS), School of Medicine from University of Minho, 4710-057, Braga, Portugal; l ICVS/3B's – PT Government Associate Laboratory, 4710-057, Braga/Guimarães, Portugal; m Department of Public Health and Forensic Sciences and Medical Education, Faculty of Medicine, University of Porto, 4200-319, Porto, Portugal; n UCIBIO - Research Unit on Applied Molecular Biosciences, Translational Toxicology Research Laboratory, University Institute of Health Sciences (1H-TOXRUN, IUCS-CESPU), 4585-116, Gandra, Portugal; o FOREN – Forensic Science Experts, Dr. Mário Moutinho Avenue, No. 33-A, 1400-136, Lisbon, Portugal

**Keywords:** High-grade serous carcinoma, Malignant ascitic fluid, Tumor microenvironment, Proteomics, Metabolic pathways

## Abstract

Ovarian cancer (OC) patients develop ascites, an accumulation of ascitic fluid in the peritoneal cavity anda sign of tumour dissemination within the peritoneal cavity. This body fluid is under-researched, mainly regarding the ascites formed during tumour progression that have no diagnostic value and, therefore, are discarded. We performed a discovery proteomics study to identify new biomarkers in the ascites supernatant of OC patients. In this preliminary study, we analyzed a small amount of OC ascites to highlight the importance of not discarding such biological material during treatment, which could be valuable for OC management. Our findings reveal that OC malignant ascitic fluid (MAF) displays a proliferative environment that promotes the growth of OC cells that shift the metabolic pathway using alternative sources of nutrients, such as the cholesterol pathway. Also, OC ascites drained from patients during treatment showed an immunosuppressive environment, with up-regulation of proteins from the signaling pathways of IL-4 and IL-13 and down-regulation from the MHC-II. This preliminary study pinpointed a new protein (Transmembrane Protein 132A) in the OC context that deserves to be better explored in a more extensive cohort of patients’ samples. The proteomic profile of MAF from OC patients provides a unique insight into the metabolic kinetics of cancer cells during disease progression, and this information can be used to develop more effective treatment strategies.

## Highlights

• The proteomic profile of ascites is a unique opportunity to track the progression of cancer cells.
• Cholesterol pathway is increased in ascites during chemotherapy.
• Chemotherapy increases IL-4 and IL-13 pathways and decreases MHC-II in ascites.
• Ascites proteomics revealed the presence of TMEM132A protein in the context of ovarian cancer.

## Graphical abstract

Occurrence of malignant ascitic fluid (MAF) during ovarian cancer (OC) progression. In this study, we analyzed the supernatant of MAF samples drained at the time of diagnosis (naïve) and during carboplatin and paclitaxel chemotherapy. These samples were analyzed by liquid chromatography-mass spectrometry (LC/MS) and scrutinized by Proteome Discoverer 2.5.0.400 Software. Our findings evidenced a shift in the proteomic profile of MAF during disease progression. Figure created in Bionrender.com. LC/MS - liquid chromatography-mass spectrometry; APOC2 - Apolipoprotein C-II; UBA1 - Ubiquitin-like modifier-activating enzyme 1.

**Figure:**
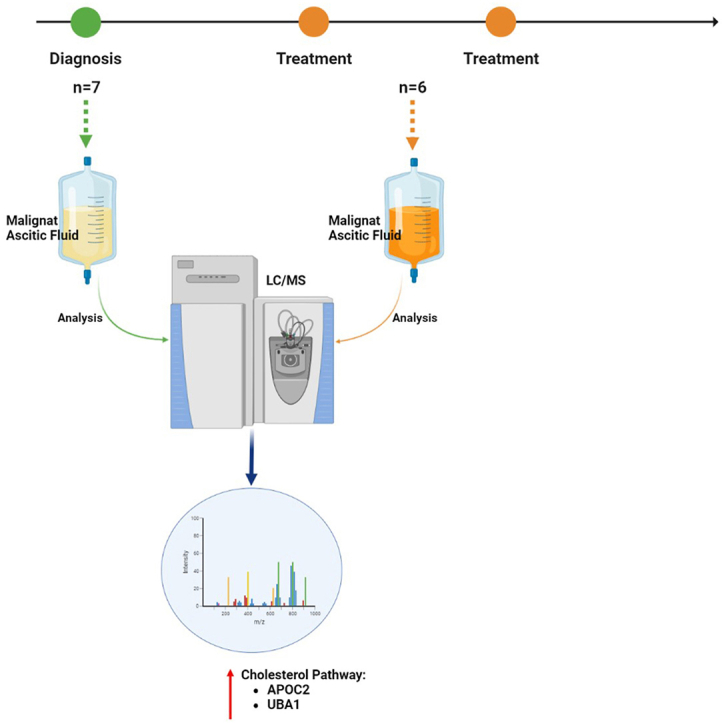

## Abbreviations

OC: Ovarian Cancer
MAF: Malignant ascitic fluid
HGSC: High-grade serous carcinoma
LC/MS: Liquid Chromatography – Mass Spectrometry
APOC2: Apolipoprotein C-II
UBA1: Ubiquitin-like modifier-activating enzyme 1
MHC-II: Major Histocompatibility Complex Class II
TMEM: Transmembrane Protein
IL: Interleukin
CAN: Acetonitrile
FA: Formic Acid
CA125: Cancer Antigen 125
HE4: Epididymis protein-4

## 1 Introduction

Ovarian cancer (OC) is one of the most frequent gynecologic cancers, and although less prevalent than breast cancer, it is three times more lethal [1]. High-grade serous carcinoma (HGSC) is the most common ovarian malignancy and is characterized by rapid growth and intraperitoneal spread [2]. Currently, screening techniques are unsuccessful for early detection of OC, and most patients (∼90 %) are diagnosed at advanced stages (III/IV) and frequently present ascites [3]. Symptoms associated with the presence of an accumulation of ascitic fluid in the peritoneal cavity (ascites) are intestinal obstruction, including nausea, vomiting, persistent bloating, and abdominal pain [4]. Available screening methods include transvaginal ultrasound, and blood biomarker analyses, such as cancer antigen 125 (CA125), epididymis protein-4 (HE4), and the OVA1 multiparametric test [5], which is a combination of five immunoassays (CA125, β2-microglobulin, transferrin, apolipoprotein A1 and pre-albumin) [5]. Typically, HGSC patients present bilateral ovarian involvement with diffuse and extensive peritoneal carcinomatosis at diagnosis [6,7]. Standard treatment for OC patients is based on cytoreductive surgery followed by platinum-taxane combination chemotherapy [8]. The response rate to first-line therapy is around 80–90 %, but most patients relapse and develop chemoresistance, contributing to a poor 5-year survival rate of <35 % [9]. MAF is a vehicle that enables the dissemination and adhesion of cancer cells in the omentum and serous membranes lining the peritoneal organs [9]. Accumulation of MAF is a multifactorial process that includes lymph obstruction, increased vascular permeability due to the secretion of growth factors, and the transition of epithelial cells to a more mesenchymal phenotype [10,11]. It is now widely accepted that MAF provides a nurturing environment for cancer progression, facilitates the metastatic process, and contributes to chemoresistance and recurrence [10,[12], [13], [14]]. MAF produces a unique microenvironment capable of modifying the neoplastic properties of tumour cells [15], and it is an extraordinary source of biological material for research due to easy accessibility, repeated collection, capacity for reflecting primary tumour, metastatic implants, and tumour microenvironment [8].

In clinical practice, OC MAF drained during disease progression is discarded, but this body fluid constitutes a unique opportunity to find new biomarkers capable of predicting therapy response. The aim of this study is to unveil some metabolic pathways and specific proteins that could be used for these purposes. To this aim, we analyzed the proteomic profile of the MAF supernatant (acellular component) and compared it at different stages of the disease progression. Our results showed that the proteins present in the MAF supernatant reflect a shift in the metabolism during disease progression and unveil some key molecules as putative therapy targets.

## 2 Material and methods

### 2.1 Samples collection

Thirteen MAF samples were collected from nine patients diagnosed with HGSC (see Supplementary Table 1) according to local ethical guidelines (as stipulated by the Declaration of Helsinki) and approved by the Ethical Committee from CES Portuguese Institute of Oncology from Porto (IPO-Porto, Ref.92R1/019). Pathologists determined the histopathological classification based on histological samples as part of the clinical diagnosis. MAF samples vary in volume (from 500 mL to 4L) and were obtained from patients at diagnosis (i.e. patients not submitted to treatment - naïve) (n = 7) and ascites retrieved from patients during chemotherapy (n = 6). The antineoplastic agents and the number of chemotherapeutic cycles administered to patients varied from between patients, but for this study, we selected only the samples obtained from patients treated with carboplatin and paclitaxel.

### 2.2 Collection of MAF samples and separating the acellular of the cellular fraction

The MAF samples were collected by the technicians of IPO-Porto and transported to i3S laboratory in Porto, Portugal. The transport of the samples was done in order to ensure their quality. Cellular and acellular fractions of MAF samples were separated by centrifugation at 400*g* for 10 min. The acellular fraction (supernatant) was stored at −80 °C, for the proteomic study. The cellular components were submitted to other proceedings and stored at −80 °C for other studies (not described here).

### 2.3 Proteomic analysis

To identify different proteins in the acellular fraction of MAF naïve samples (n = 7) and MAF during therapy samples (n = 6), we utilized the Pierce™ BCA Protein Assay Kit (ref. 23225, Thermo Scientific) for protein detection and quantification. Subsequently, all samples were subjected to LC-MS/MS analysis. This analysis was conducted using an Ultimate 3000 liquid chromatography system coupled to a Q-Exactive Hybrid Quadrupole-Orbitrap mass spectrometer (Thermo Scientific, Bremen, Germany). For sample preparation, 500 ng of peptides from each sample were loaded onto a trapping cartridge (Acclaim PepMap C18 100 Å, 5 mm × 300 μm i.d., 160454, Thermo Scientific, Bremen, Germany) at a flow rate of 10 μL/min, using a mobile phase consisting of 2 % acetonitrile (CAN) and 0.1 % formic acid (FA). After 3 min of loading, the trap column was switched in-line to a 50 cm × 75 μm inner diameter EASY-Spray column (ES803, PepMap RSLC, C18, 2 μm, Thermo Scientific, Bremen, Germany) at 250 nL/min. Separation was achieved by a gradient of solvent A (0.1 % FA) and solvent B (80 % CAN, 0.1 % FA) with the following gradient profile: 5 min (2.5 % B to 10 % B), 120 min (10 % B to 30 % B), 20 min (30 % B to 50 % B), 5 min (50 % B to 99 % B), followed by 10 min (hold 99 % B). The column was then equilibrated with 2.5 % B for 17 min. Data acquisition was controlled by Xcalibur 4.0 and Tune 2.9 software (Thermo Scientific, Bremen, Germany). The mass spectrometer operated in the data-dependent (dd) positive acquisition mode, alternating between a full scan (*m*/*z* 380–1580) and subsequent HCD MS/MS of the 10 most intense peaks from a full scan, with a normalized collision energy of 27 %. The ESI spray voltage was set at 1.9 kV. Global settings included the use of lock masses (*m*/*z* 445.12003), lock mass injection Full MS, and chromatographic peak width (FWHM) of 15 s. Full scan settings were as follows: 70 k resolution (*m*/*z* 200), AGC target 3 × 10^6^, and a maximum injection time of 120 ms. For dd settings, the minimum AGC target was 8 × 10^3^, the intensity threshold was 7.3 × 10^4^, and charge exclusion included unassigned, 1, 8, and >8 charges, with peptide match preferred and isotopes excluded. Dynamic exclusion was set to 45 s. MS2 settings were as follows: microscans 1, resolution 35 k (*m*/*z* 200), AGC target 2 × 10^5^, maximum injection time 110 ms, isolation window 2.0 *m*/*z*, isolation offset 0.0 *m*/*z*, dynamic first mass, and spectrum data type profile.

### 2.4 Database searching and protein identification

The raw data were processed using the Proteome Discoverer 2.5.0.400 software (Thermo Scientific, Bremen, Germany). Protein identification analysis was performed with the data available in the UniProt protein sequence database for the Homo sapiens Proteome 2020_05 with 75,069 entries and a common contaminant database from MaxQuant (version 1.6.2.6, Max Planck Institute of Biochemistry, Munich, Germany) [15]. Additionally, before analysis, rigorous measures were taken to exclude contaminants and reverse identifications from the dataset, ensuring the integrity of the subsequent analysis. Protein identifications were subjected to stringent criteria, requiring a probability threshold of 99 % or higher, along with the detection of a minimum of two unique peptides. These criteria were chosen deliberately to support a high standard of confidence and to minimize false discoveries, with the false discovery rate typically maintained at less than 1 % using a decoy database search strategy at the protein level. Furthermore, in cases where proteins contained similar peptides that could not be distinguished based exclusively on MS/MS analysis, a parsimonious approach was adopted, grouping these proteins together to avoid redundancy and restructure the analysis process. This meticulous approach to data organization and analysis was crucial for generating meaningful insights and drawing valid conclusions from the experimental results.

### 2.5 Statistical analysis

**Fig. 1:**
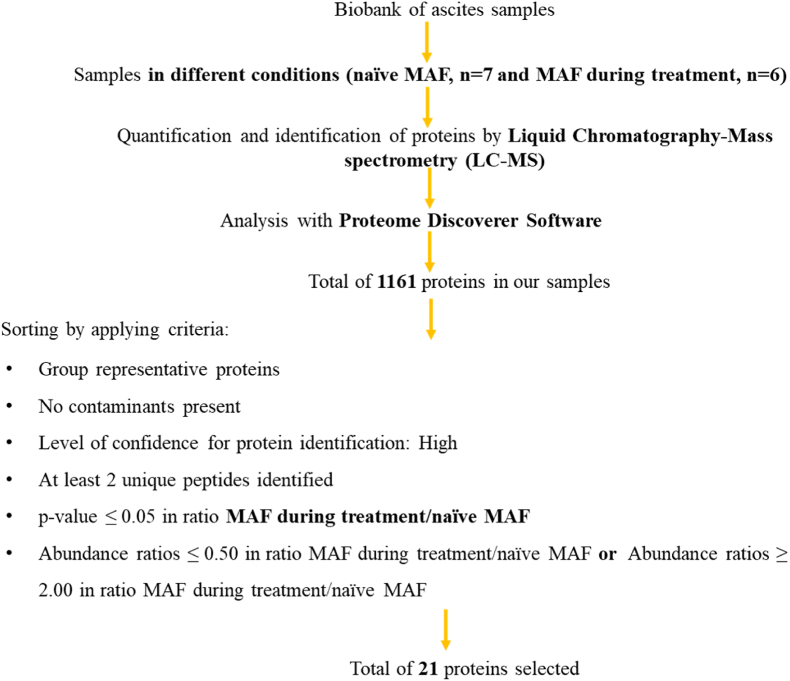
Pipeline used to select proteins that are differentially expressed in supernatant of ascitic fluid samples. MAF – malignant ascitic fluid.

Student's t-test was used for statistical analysis, which involved comparing two groups (MAF naïve/MAF during therapy). Unless otherwise stated, data were presented as mean ± SEM (n = 3 biological replicates for each sample), with *p < 0.05 considered statistically significant, by the p-value adjusted using Benjamin-Hochberg correction for the false-discovery rate. The abundance ratio reflects the protein abundance and was calculated from the sum of all unique normalized peptide ion abundances for a specific protein on each run. Data was analyzed using the Proteome Discoverer 2.5.0.400 software and using the pipeline described in Fig. 1, allowing the identification of the proteins differentially expressed at different conditions.

### 2.6 Immunocytochemistry

In supplementary data, immunocytochemistry (ICC) of cellular components obtained from the MAF of HGSC patient 12 (HGSC#12) was performed using a manual system. After deparaffinization and hydration, heat-induced antigen retrieval was conducted at 98 °C using either citrate buffer solution (1:100 at pH 6.0; ThermoFisher Scientific, Waltham, MA, USA) or ethylenediaminetetraacetic acid (EDTA; 1:100; ThermoFisher Scientific, Waltham, MA, USA). The Dako REAL™ EnVision™ Detection System Peroxidase/DAB+, Rabbit/Mouse (Agilent Dako, Santa Clara, CA, USA) kit was employed for detection according to the manufacturer's instructions. Initially, endogenous peroxidase activity was blocked with a 3 % (v/v) hydrogen peroxide solution (ThermoFisher Scientific, Waltham, MA, USA). Immunostaining was carried out using monoclonal antibodies for ALDH1 (1:200, D9Q8E, Cell Signaling Technology, Massachusetts, MA, USA) and SOX2 (1:25, SP76, Cell Marque, California, CA, USA), with an incubation period of 1 h at room temperature. Primary antibodies were then detected using a secondary antibody labelled with horseradish peroxidase (HRP)-conjugated polymer, followed by visualization of the reaction using diaminobenzidine, as the manufacturer's instructions. Subsequently, nuclear staining was performed with hematoxylin, and slides were dehydrated, clarified, and sealed with coverslips using a permanent mounting medium for optical microscope analysis. ICC results were evaluated by three independent observers (S.R., M.N. and D.N.).

## 3 Results

**Fig. 2:**
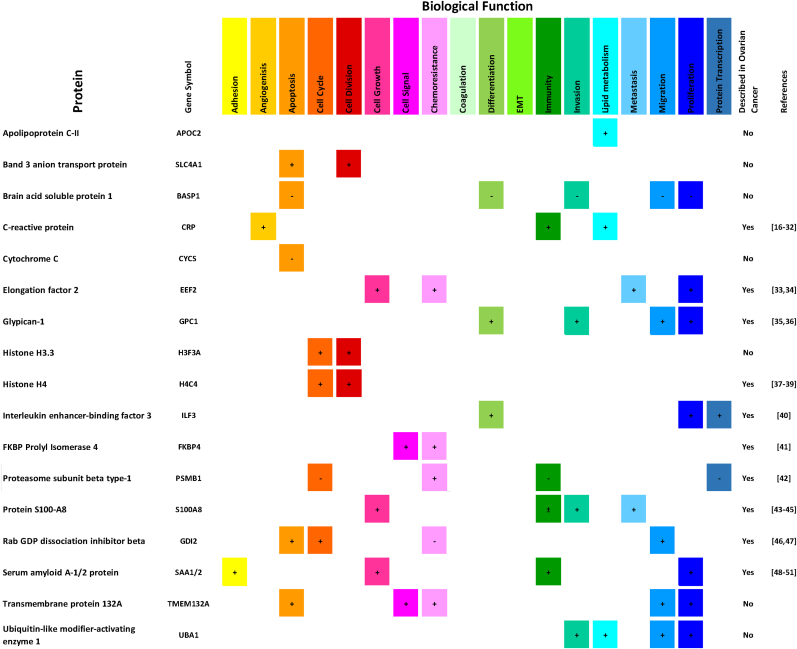
Summary of the pro-tumoral and anti-tumoral relevant proteins (n = 18) present in all MAF samples of OC patients. Proteins described as pro-tumoral (+), anti-tumoral (−); and promoters or suppressors of tumorigenesis according to the tumor context (±).

After analyzing the samples from OC patients at diagnosis and during treatment, we selected 18 expressed proteins in all MAF samples analyzed (n = 13). This selection excluded the immunoglobulins and hemoglobin as a direct consequence of the presence of blood in ascites. Then, we grouped these proteins based on their biological function and investigated if they had been previously described in OC [[16], [17], [18], [19], [20], [21], [22], [23], [24], [25], [26], [27], [28], [29], [30], [31], [32], [33], [34], [35], [36], [37], [38], [39], [40], [41], [42], [43], [44], [45], [46], [47], [48], [49], [50], [51]]. The selected 18 proteins, from the 13 MAF samples, were described as pro-tumoral or anti-tumoral (represented by the symbol + and -, respectively) (Fig. 2). Furthermore, proteins were categorized based on their biological functions, including cell adhesion, angiogenesis, apoptosis, cell cycle, cell division, cell growth, cell signaling, chemoresistance, coagulation, differentiation, epithelial-mesenchymal transition (EMT), immunity, invasion, lipid metabolism, metastasis, migration, proliferation, and protein transcription. Our investigation affirms that a majority of the proteins identified in the MAF supernatant serve a pro-tumor role, participating in various stages of tumor progression such as cell proliferation, migration, and invasion. Additionally, our proteomic analysis highlights a metabolic shift towards lipid metabolism that promotes cancer progression. These findings suggest that targeting this metabolic adaptation could be a promising approach in cancer treatment.

### 3.1 Proteomic modifications in the MAF during chemotherapy

**Fig. 3:**
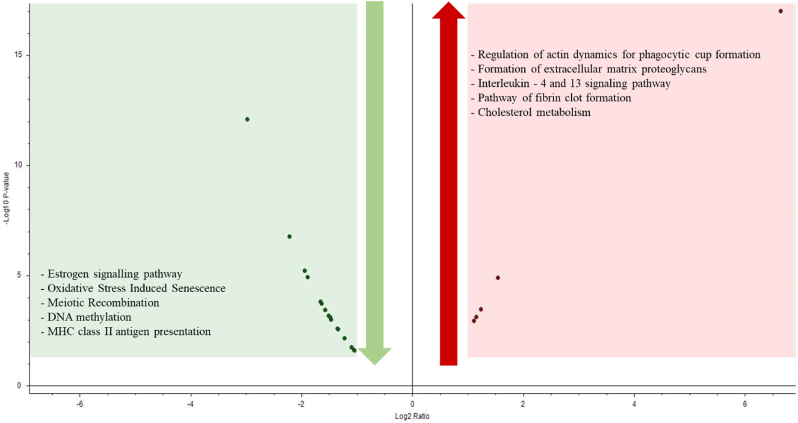
Volcano plot representing the variation of proteins from different pathways comparing MAF during treatment vs naïve MAF samples. The top 5 pathways downregulated (green box) and up-regulated (red box) are also described in the volcano plot. These enrichment data were obtained from Proteome Discoverer 2.5.0.400 Software by Gene ontology enrichment, based on the total number of proteins identified (n = 1161). (For interpretation of the references to color in this figure legend, the reader is referred to the Web version of this article.)

In OC patients with ascites, the liquid tumoral microenvironment can persist or arise during chemotherapy. The MAF can provide insight into the effect of antineoplastic drugs on the tumor. To this end, we analyzed 7 naïve MAF (obtained at diagnosis) and 6 MAF drained during chemotherapy. Our aim was to compare the MAF at these two points of the disease and understand the effect of chemotherapy in the metastatic milieu. We examined a total of 1161 proteins (see the list in Supplementary Table 2) present in the 13 samples and selected 21 based on our statistical criteria, although 3 of them were excluded because were immunoglobulins and/or hemoglobin. These proteins are associated with important biological processes such as stress response, protein metabolism, cell organization and biogenesis, signal transduction, and cell proliferation. The proteins selected can provide insights into the effectiveness of chemotherapy in the liquid tumoral microenvironment. For more details on the proteins selected, please refer to Supplementary Table 3 and Fig. 1. Fig. 3 shows a volcano plot of the proteins increased and decreased, as well as the top 5 pathways, up and downregulated when compared MAF during treatment with naïve MAF.

**Fig. 4:**
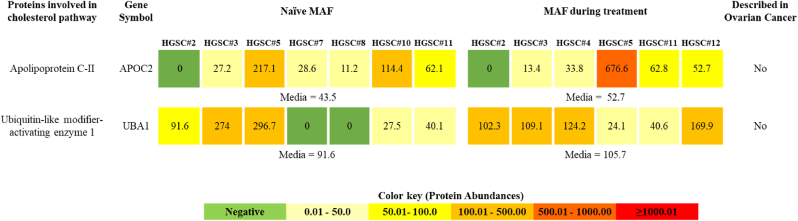
Heatmap of proteins abundances related to cholesterol pathway, found in naïve and during treatment MAF samples. Data obtained from Proteome Discoverer 2.5.0.400 Software. Protein abundance was calculated from the sum of all unique normalized peptide ion abundances for a specific protein on each run.

Gene ontology (GO) enrichment analysis identified the regulation of actin dynamics for phagocytic cup formation, extracellular matrix proteoglycans formation, interleukin-4 and 13 signalling pathway, fibrin clot formation pathway, and cholesterol metabolism as the top 5 upregulated pathways in MAF during treatment (Fig. 3). On the other hand, the estrogen signalling pathway, oxidative stress that triggers cellular senescence, meiotic recombination, DNA methylation, and major histocompatibility complex class II (MHC-II) antigen presentation were among the most downregulated pathways (Fig. 3). Our data indicates that there is a metabolic shift in the MAF acellular fraction when treated with carboplatin and paclitaxel, which leads to an increased expression of two proteins that are intrinsic to the cholesterol pathway. The abundance of these cholesterol pathway proteins in naïve MAF and during treatment is depicted in Fig. 4. Specifically, we found that apolipoprotein C-II and ubiquitin-like modifier-activating enzyme 1 were more abundantly expressed in MAF obtained during therapy.

Our analysis of protein abundances in the same patient with both time-point collection samples (naïve and during treatment), specifically HGSC#2, HGSC#3, HGSC#5, and HGSC#11, confirmed our findings when comparing disease progression at two different time points. Additional data supporting our study is available in Supplementary Tables 4, 5, 6, and 7.

Interestingly, we observed that the transmembrane protein 132A (TMEM132A) was highly expressed in one MAF sample from a patient with platinum resistance. This should be better explored in a cohort of patients with the same chemoresistance profile to better understand the link between this specific protein and putative association to chemoresistance profile.

## 4 Discussion

The microenvironment surrounding MAF is rich in proteins that promote tumor growth, progression, and metastasis [52]. Our study compared MAF samples obtained before and during treatment, and found that the estrogen pathway is suppressed in samples obtained during treatment. Estrogen signals through estrogen receptor α (ERα) by activating it in the cytoplasm through E2, leading to ERα dimerization in the nucleus and direct/indirect DNA binding for regulating genes involved in the cell cycle, cell migration, proliferation, and differentiation [53,54]. ERα and ERβ receptors are expressed in various cancer types, including OC, but their signalling pathways have different effects. Estrogen also plays a role in the PI3K/AKT pathway, which mediates the ERBB2/ERBB3 oncogenic signalling pathway that promotes tumour development and progression [55]. While the exact mechanism by which chemotherapy blocks the estrogen pathway needs further study, it is thought to involve the interference of enzymes and other molecules involved in hormone production and signalling.

OC progression has been linked with active expression of aerobic glycolysis or oxidative phosphorylation (OXPHOS) and abnormal lipid metabolism [56]. Also, MAF supernatant is recognized as an adipocyte and lipid-rich environment that contributes to tumor development, immune evasion, chemoresistance, and recurrence [57,58]. Another proteomic study on liquid ascitic extracellular vesicles describes them as a biological sample rich in lipoproteins [59]. Our proteomic analysis indicated an increase in the expression of several proteins of the cholesterol metabolism pathway in MAF samples. Certain enzymes that participate in the metabolic processes of cholesterol have been found to support the advancement of tumour cells, promote resistance to chemotherapy treatments, and create an environment within the tumour that suppresses the immune system [[55], [56], [57]]. This liquid tumour environment is of great significance to be used as a medium supplementation in patient-derived cultures, creating a more accurate replication of patient tumour conditions. Additionally, MAF samples obtained during treatment showed an increased abundance of apolipoprotein C-II and ubiquitin-like modifier-activating enzyme 1 (UBA1) (Fig. 4). Apolipoprotein C-II regulates lipid metabolism, and its interaction with CD36 promotes tumour progression via PI3K/AKT/mTOR signalling, which regulates the EMT process [60]. UBA1 is a protein that activates a signalling pathway called Nrf2, which plays a crucial role in regulating cell proliferation, invasion, migration, and ferroptosis [61]. Ferroptosis is a type of iron-dependent cell death that leads to the accumulation of ferric iron and promotes lipid peroxidation. UBA1 is also involved in cholesterol metabolism through ferroptosis [61]. Growing evidence suggests that changes in lipid metabolism are closely associated with drug resistance in tumours. Identifying the proteins that play a role in this metabolic shift and developing therapeutic strategies that combine lipid metabolism-targeted therapies with traditional chemotherapy drugs may help overcome drug resistance and improve OC patient's survival.

One interesting result was the identification of highly expressed levels of TMEM132A in one specific sample retrieved from a patient that was not responsive to platinum therapy. TMEMs are a family of proteins that span the entire width of the cellular lipid bilayer. These proteins are permanently attached to the membrane and act as channels to transport specific substances across various biological membranes, such as mitochondrial, endoplasmic reticulum, lysosome, and Golgi membranes [62]. TMEMs are a family of proteins that have been found to be expressed in various cancers, including lymphomas (TMEM176) [63], colorectal (TMEM25) [64], hepatic (TMEM7) [65], and lung cancers (TMEM48) [66]. Some proteins of this family are potentially useful for classifying cancer grade in renal cancers (e.g., TMEM45A, TMEM116, TMEM207, TMEM213) [67]. They also have been related to cancer development and drug resistance, suggesting that the TMEM family is a major group for cancer research. Furthermore, some of these proteins act as tumor suppressors (e.g., TMEM25, TMEM7) [68] while others act as pro-oncogenes (e.g., TMEM158, TMEM14A) [69]. The TMEM protein family has a crucial role in cancer research and drug development, as shown by the evidence. This study is the first to describe TMEM132A in the context of OC, and further research is needed to determine its role in this particular body fluid. We found that TMEM132A plays an oncogenic role in regulating cell proliferation, migration, and invasion in gastric cancer [70]. This intriguing result in this particular MAF sample should be further investigated in the context of a chemoresistance profile since, to the best of our knowledge, it has not been described in the OC context so far. Tumor cells in the MAF of this particular patient showed a molecular profile associated with chemoresistance, i.e. with high expression levels of SOX2 and ALDH1 measured by immunocytochemistry (see Supplementary Fig. 1). Cancer stem-like cells represent a small subpopulation of undifferentiated cells capable of self-renewal, proliferation, differentiation, and multipotency, and also are related to disease relapse and treatment resistance [71]. An enlarged series of MAF cases will be crucial to better explore the correlation between the chemoresistance profile in tumour cells and the abundance of such proteins in OC MAF samples. In MAF drained during treatment, proteins involved in Interleukin (IL)-4 and −13 signalling pathways are increased. The OC has a tumour microenvironment that is immunosuppressive and has high levels of cytokines like IL-4 and IL-13 that promote the recruitment of immunosuppressive cells [72]. IL-4 and IL-13 are two closely related cytokines that have pleiotropic effects on immune responses and the microenvironment, in both normal and cancerous conditions [73]. These cytokines are mostly produced by T helper 2 (Th2) cells, as well as macrophages, dendritic cells, and natural killer (NK) cells [74]. They are also important regulatory cytokines in the tumor microenvironment, activating tumor-associated macrophages and myeloid-derived suppressor cells (MDSC) that support pro-tumor activity [72,75,76]. IL-4 and -13 receptors are up-regulated and activated in many tumours, including OC [[77], [78], [79], [80]], which initiates the phosphorylation of Stat6, leading to increased tumour cell proliferation and resistance to apoptosis [81]. The microenvironment of MAF contains a high concentration of cytokines, which play a crucial role in transmitting abnormal cellular signals through various pathways, ultimately leading to the aggressive behaviour of OC. The identification of these interleukins in MAF could be useful in identifying tumours with more aggressive profiles.

Our research has revealed that OC MAF obtained during treatment exhibits low levels of MHC-II expression. MHC-II molecules are commonly expressed by antigen-presenting cells (APC), such as dendritic and B cells, and macrophages that interact with and activate CD4^+^ T cell responses. CD4^+^ T cells play a crucial role in activating CD8^+^ T cells, which are essential for an effective response to immune checkpoint inhibitors [[82], [83], [84], [85]]. Tumor-specific MHC-II expression has been linked to a favorable prognosis and improved response to immune checkpoint inhibitors [[86], [87], [88], [89], [90]]. Therefore, the low MHC-II levels found in MAF drained during treatment could serve as an indicator of patient prognosis and valuable information to identify patients who may benefit from immunotherapy.

## 5 Conclusions

This study used a discovery proteomics approach to identify new biomarkers in the MAF supernatant of OC patients. Although a low number of samples were analyzed, it is important to note that MAF drained during treatment are usually discarded. Therefore, this study emphasizes the significance of not discarding such biological material, which could be valuable for managing OC.

Our findings reveal that OC MAF displays a proliferative environment that promotes the growth of OC cells that shift the metabolic pathway using alternative sources of nutrients, such as the cholesterol pathway. These findings suggest using drugs to block the proteins involved in this pathway to block cancer cell growth. Our analysis of OC MAF samples from patients during treatment also showed an immunosuppressive environment, with up-regulation of signalling pathways of IL-4 and IL-13 and down-regulation of MHC-II. This information can be used to develop targeted immunotherapies to improve patient outcomes. The OC MAF samples used in this study offer a precious source of information for longitudinal studies the OC setting where the biological material obtained during disease progression is limited or difficult to obtain. The proteomic profile of MAF from OC patients provides a unique insight into the metabolic kinetics of cancer cells during disease progression, and this information can be used to develop more effective treatment strategies in the near future.

## Consent for publication

All authors agree with the content of the manuscript.

## Data Availability statement

The data presented in this study are available in this article and Supplementary Material.

## Institutional review board statement

The study was conducted in accordance with the Declaration of Helsinki and approved by the Ethical Committee from CES Portuguese Oncology Institute of Porto (IPO-PORTO, Ref.92R1/019). The informed consent of all patients was obtained for this study.

## Funding

This work was developed at i3S/IPATIMUP, an Associate Laboratory of the Portuguese Ministry of Science, Technology and Higher Education, and partially supported by Fundacao para a Ciencia e a Tecnologia (10.13039/501100001871FCT). This research was supported by European Regional Development Funds (10.13039/501100008530ERDF) funds through the 10.13039/501100011929COMPETE 2020–Operational Program for Competitiveness and Internationalization (POCI), Portugal 2020, 10.13039/501100001871FCT/Ministério da Ciencia, Tecnologia e Inovacao (10.13039/501100006111MCTES), under the project POCI 01-0145-FEDER-029503 (PTDC/MEC-10.13039/100005982ONC/29503/2017). This work was also supported by the Portuguese Mass Spectrometry Network, integrated in the National Roadmap of Research Infrastructures of Strategic Relevance (ROTEIRO/0028/2013; LIS-BOA-01-0145-FEDER-022125). Ricardo Silvestre acknowledges to the 10.13039/501100001871FCT for financial support through CEEC contracts 10.54499/2020.00185.CEECIND/CP1600/CT0004. Diana Nunes and Mariana Nunes acknowledges 10.13039/501100001871FCT/10.13039/501100006111MCTES and União Europeia (10.13039/501100012637UE) for financial support through a PhD fellowship (2021.05081.10.13039/100017412BD and 10.13039/1000174122020.04720.BD - DOI: 10.54499/2020.04720.BD, respectively) co-sponsored by Fundo Social Europeu (FSE) through Programa Operacional Regional Norte (Norte 2020).

## CRediT authorship contribution statement

**Diana Luísa Almeida-Nunes:** Writing – original draft, Visualization, Validation, Methodology, Formal analysis, Conceptualization. **Mariana Nunes:** Writing – original draft, Validation, Methodology, Data curation. **Hugo Osório:** Validation, Methodology, Data curation. **Verónica Ferreira:** Resources, Methodology. **Cláudia Lobo:** Methodology. **Paula Monteiro:** Resources, Methodology. **Miguel Henriques Abreu:** Resources, Methodology. **Carla Bartosch:** Supervision, Resources, Methodology. **Ricardo Silvestre:** Writing – review & editing, Supervision. **Ricardo Jorge Dinis-Oliveira:** Writing – review & editing, Validation, Supervision, Investigation. **Sara Ricardo:** Writing – review & editing, Validation, Supervision, Investigation, Conceptualization.

## Declaration of competing interest

The authors declare that they have no known competing financial interests or personal relationships that could have appeared to influence the work reported in this paper.
