# Supplementary material for: Ovarian cancer ascites proteomic profile reflects metabolic changes during disease progression
Source: Biochem Biophys Rep. 2024 Jun 13;39:101755. doi: 10.1016/j.bbrep.2024.101755 (PMC11225207; doi:10.1016/j.bbrep.2024.101755)
Supplement: Multimedia component 1 [file mmc1.docx]

**Supplementary Material**

**Supplementary Table 1.** **The summary table of patient’s characteristics of the study**

| **Patients** | **Age** | **Gender** | **Histologic**  **characterization** | **Stage** | **Surgery** | **Platinum Response** | **Time-point samples collection** | | |
| --- | --- | --- | --- | --- | --- | --- | --- | --- | --- |
|  |  |  |  |  |  |  | **At Diagnosis** | **During Treatment#**  **(**Carboplatin+Paclitaxel) | **At**  **Recurrence** |
| **HGSC#2** | 76† | Female | High-Grade Serous Carcinoma | IIIC | With surgery | Sensitive | MAF collection | MAF collection | - |
| **HGSC#3** | 71† | Female | High-Grade Serous Carcinoma | IV | Without Surgery | Refractory | MAF collection | MAF collection | - |
| **HGSC#4** | 75† | Female | High-Grade Serous Carcinoma | IIIC | Without Surgery | Sensitive | - | MAF collection | - |
| **HGSC#5** | 72† | Female | High-Grade Serous Carcinoma | IV | With surgery | Sensitive | MAF collection | MAF collection | - |
| **HGSC#7** | 65† | Female | High-Grade Serous Carcinoma | IV | Without Surgery | Refractory | MAF collection | - | - |
| **HGSC#8** | 63 | Female | High-Grade Serous Carcinoma | IIIC | With surgery | Sensitive | MAF collection | - | - |
| **HGSC#10** | 89† | Female | High-Grade Serous Carcinoma | IV | Without Surgery | NA | MAF collection | - | - |
| **HGSC#11** | 68† | Female | High-Grade Serous Carcinoma | IIIC | Without Surgery | Sensitive | MAF collection | MAF collection | - |
| **HGSC#12*** | 68 | Female | High-Grade Serous Carcinoma | IIIC | With surgery | Resistant | - | - | MAF collection |
|  |  |  |  |  |  |  | MAF Naïve = 7 | MAF during treatment =6 | |

**Supplementary Table 1. The summary table of patient’s characteristics of the study.** HGSC – High-grade serous carcinoma; †Deceased patient; *Patient clinically identified as platinum resistant; # During Treatment = Neoadjuvant or palliative care; X=Collection time-point. **Note:** The majority of malignant ascitic fluid was collected during neoadjuvant chemotherapy or palliative chemotherapy


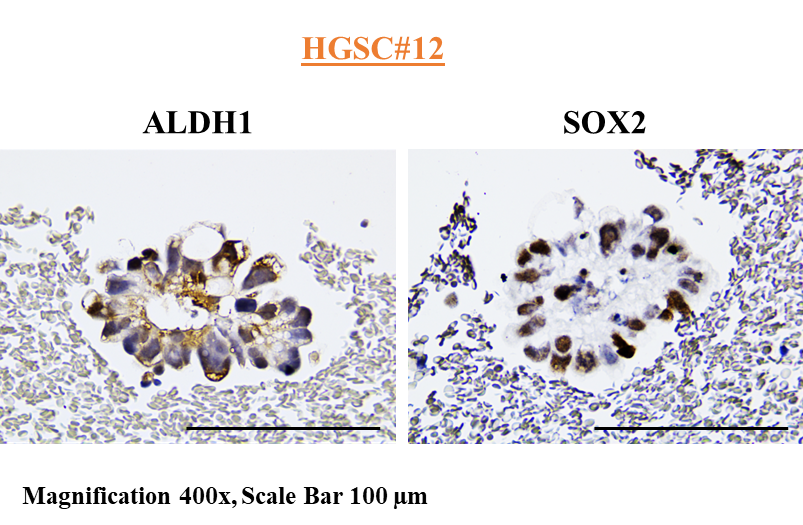


**Supplementary Figure 1: Representative immunocytochemistry images of cancer stem cell markers expression (ALDH1 – brown stain in the cytoplasm; SOX2 – brown stain in the nucleus) in tumor cells in patient HGSC#12. The proteomic analysis of the supernatant from this MAF revealed that TMEM132A was present in high abundance. All the images were taken at 400x magnification at a Brightfield Microscope Leica DM2000 LED (Leica Microsystems, Wetzlar, Germany). The scale bar represents 100 μm.**
